# The deubiquitinating enzyme STAMBP is a newly discovered driver of triple-negative breast cancer progression that maintains RAI14 protein stability

**DOI:** 10.1038/s12276-022-00890-1

**Published:** 2022-11-25

**Authors:** Qianqian Yang, Ding Yan, Chaoying Zou, Qian Xue, Shuhui Lin, Qingtian Huang, Xiaofen Li, Daolin Tang, Xin Chen, Jinbao Liu

**Affiliations:** 1 Affiliated Cancer Hospital & Institute of Guangzhou Medical University, Guangzhou, 510095 China; 2 Guangzhou Municipal and Guangdong Provincial Key Laboratory of Protein Modification and Degradation, State Key Laboratory of Respiratory Disease, School of Basic Medical Sciences, Guangzhou Medical University, Guangzhou, 511436 China; 3 Department of Surgery, UT Southwestern Medical Center, Dallas, TX 75390 USA

**Keywords:** Cancer, Cancer

## Abstract

Triple-negative breast cancer (TNBC) is a heterogeneous malignancy in women. It is associated with poor prognosis, aggressive malignant behavior, and limited treatment options. In the ubiquitin‒proteasome system (UPS), deubiquitinases (DUBs) are potential therapeutic targets for various tumors. In this study, by performing unbiased siRNA screening, we identified STAMBP, a JAMM metalloprotease in the DUB family, as a driver of human TNBC tumor growth. Functionally, the knockdown of STAMBP inhibited the proliferation, migration, and invasion of multiple TNBC cell lines. Immunoprecipitation–mass spectrometry combined with functional and morphological analysis verified the interaction between STAMBP and the actin-binding protein RAI14. Mechanistically, STAMBP stabilized the RAI14 protein by suppressing the K48-linked ubiquitination of RAI14 and thus prevented its proteasomal degradation. Therefore, knocking down STAMBP resulted in the reduction in RAI14 protein levels and suppression of tumor growth in vitro and in vivo. Importantly, high levels of STAMBP were correlated with poor prognosis in TNBC patients. In summary, we reveal a previously unrecognized DUB pathway that promotes TNBC progression and provides a rationale for potential therapeutic interventions for the treatment of TNBC.

## Breast cancer: Novel therapeutic target identified for triple-negative subtype

An enzyme that promotes tumor growth in triple-negative breast cancer (TNBC), one of the five main breast cancer subtypes, could provide a novel treatment target for this aggressive, life-limiting disease. There are limited treatment options for TNBC, and tumors often develop resistance to chemotherapy. Now, Xin Chen and Jinbao Liu at the Guangzhou Medical University, China, and co-workers have identified a key deubiquitinating enzyme involved in tumor growth in TNBC. High levels of this enzyme, the STAM binding protein (STAMBP), are associated with poor prognosis in patients. In experiments on human cell cultures and a mouse model, the team found that STAMBP stabilizes a particular protein, RAI14, that facilitates the growth of TNBC cells. Knocking out STAMBP reduced levels of this protein, consequently inhibiting cell proliferation, migration, and invasion.

## Introduction

Breast cancer is the most commonly diagnosed malignancy and one of the leading causes of cancer-related deaths in women worldwide^1^. As a highly heterogeneous disease, breast cancer can be classified into five subtypes based on its genetic and histopathological features^2^. These types include basal-like/triple-negative breast cancer (TNBC), luminal A, luminal B, human epidermal growth factor receptor 2 (HER2)-enriched, and normal-like breast cancer. TNBC is characterized by tumors that do not express estrogen receptor (ER), progesterone receptor (PR), or HER2 genes and accounts for approximately 15% of all breast cancer cases^3^. As an aggressive and metastatic subtype, TNBC lacks a recognized molecular target and often develops resistance to chemotherapy^4^. Thus, the exploration of novel targets for the treatment of TNBC is urgently needed.

The ubiquitin‒proteasome system (UPS) is a major intracellular protein quality control pathway in all eukaryotes. Ubiquitination is a dynamic process catalyzed by sequential reactions involving an E1 activating enzyme, an E2 conjugating enzyme, and an E3 ligase^5^. It is involved in the regulation of many central biological processes, influencing the stabilization, function, and subcellular localization of substrate proteins^6,7^. Ubiquitin carries seven lysine residues (K6, K11, K27, K29, K33, K48, and K63), and each residue can participate in sequential ubiquitination to generate different forms of polyubiquitination chains. Under normal conditions, ubiquitination is a reversible process regulated by deubiquitinase (DUBs). DUBs remove ubiquitin from substrate proteins and protect proteins from proteasomal degradation, thereby enhancing protein stability. Under pathological conditions, including cancer, dysfunctional DUBs negatively affect the UPS, thereby increasing protein stability and aggregation^8^. Recent studies have linked DUBs to the development of TNBC. For instance, USP28 promoted the growth of TNBC cells by regulating a DNA damage checkpoint^9^, while MYSM1 sensitized TNBC cells to cisplatin treatment^10^. In addition, USP2 maintained the self-renewal, expansion and chemoresistance of TNBC stem cells^11^. Accordingly, pharmacological inhibition of DUB activity holds promise for treating cancers, including TNBC.

The classical DUBs can be categorized into six subclasses: ubiquitin C-terminal hydrolases (UCHs), ubiquitin-specific proteases (USPs), ovarian-tumor proteases (OTUs), Machado–Joseph disease protein domain proteases (MJDs), the monocyte chemotactic protein-induced protein (MCPIP), and Zn-dependent JAMM/MPN domain–associated metallopeptidases (JAMMs)^12^. Among the DUBs, STAM-binding protein (STAMBP) belongs to the JAMM metalloprotease subfamily. STAMBP acts as an endosome-associated DUB by interacting with the SH3 domain of STAM, a component in the endosomal sorting complexes required for transport (ESCRT) machinery^13^. STAMBP mutation causes microcephaly–capillary malformation syndrome, which is associated with elevated levels of ubiquitin protein aggregates^14^. STAMBP promotes inflammasome activity in innate immune responses by mediating the deubiquitination of NALP7 and preventing the transport of NALP7 to lysosomes, thereby increasing NALP7 abundance^15^. STAMBP also inhibits excessive activation of the inflammasome by preventing the nondegradative ubiquitination of NLRP3^16^. STAMBP may promote the stabilization of Unc-51-like autophagy-activating kinase 1 (ULK1) by removing its K48-linked ubiquitin chains, thereby participating in the regulation of autophagic flux^17^. Although STAMBP has been implicated in the tumor metastasis of lung adenocarcinoma and melanoma^18,19^, the pathological role of STAMBP in TNBC has not been elucidated.

In this study, we identified a previously unrecognized role of STAMBP in regulating the proteasomal degradation of retinoic acid-induced 14 (RAI14; an actin-binding protein) in human TNBC cells. STAMBP favors the growth of TNBC by removing the ubiquitination of RAI14 and enhancing its stability in vitro and in vivo. In a clinical setting, overexpressed STAMBP was associated with poor clinical outcomes in TNBC. Hence, our data provide the first evidence for the pathological role of STAMBP in TNBC.

## Materials and methods

### Cell lines and cell culture

The MDA-MB-231 and BT549 human breast cancer cell lines were purchased from the American Type Culture Collection (ATCC; Manassas VA, USA). The MDA-MB-231 and BT549 cells were cultured in DMEM/F12 and DMEM, respectively, with 10% fetal bovine serum (FBS). These cell lines were grown in an incubator maintained at 37 °C with 5% CO_2_. All the cells were mycoplasma-free and authenticated using short tandem repeat DNA profiling analysis.

### Reagents and primary antibodies

Bafilomycin A1 (S1413), cycloheximide (S7418), and MG132 (S2619) were purchased from Selleck Chem (Houston, TX, USA). Anti-STAMBP (sc-271641) and anti-ubiquitin (sc-8017) antibodies were purchased from Santa Cruz Biotechnology (CA, USA). Anti-RAI14 (ab137118), anti-MMP9 (ab137867), and anti-PABPC1 (10970-1-AP) antibodies were purchased from Abcam (USA). Anti-RBM14 (10196-1-AP) and anti-LIMA1 (16639-1-AP) antibodies were purchased from Proteintech Group (Chicago, USA). An anti-ki67 (BS1454) antibody was purchased from Bioworld Technology (Louis Park, MN, USA). The following antibodies were obtained from Cell Signaling Technology (Beverly, MA, USA): anti-K48-ub (12805), anti-HA-tag (3724S), anti-DYKDDDDK (FLAG) tag (14793), E-Cadherin (3195T), Slug (9585T), Vimentin (5741), and anti-GAPDH (5174) antibodies.

### siRNA transfection

The siGENOME SMARTpool siRNA Library of human deubiquitinating enzymes was obtained from Dharmacon-Horizon Discovery (Cambridge, England). RAI14 siRNAs were purchased from GenePharma (Shanghai, China). The sequences of the RAI14 siRNAs were as follows (all are shown in the 5′–3′ orientation): RAI14 siRNA-1: sense: GGAUGUGACAGCCCAAGAUtt, anti-sense: AUCUUGGGCUGUCACAUCCtt; and RAI14 siRNA-2: sense: GCUGAUAGCUUAUUGGAUAtt, anti-sense: UAUCCAAUAAGCUAUCAGCtt. Exponentially proliferating cells were seeded in dishes or well plates for 24 h. siRNAs (50 nM) were mixed with Lipofectamine 3000 transfection reagent according to the manufacturer’s instructions (Invitrogen, L3000015). After 15 min of incubation, this mixture was added to each group. The cells were cultured for the indicated times before analysis.

### Lentiviral shRNA transfection

Lentivirus (hU6-MCS-Ubiquitin-firefly_Luciferase-IRES-puromycin) containing two pairs of shRNAs targeting STAMBP or nonspecific sequences (control shRNAs) were purchased from GeneChem (Shanghai, China). The sequences of the STAMBP shRNAs were as follows (all are shown in 5′–3′ orientation): STAMBP shRNA-1: sense: CCUUCAUCCUCUAUAACAAtt, antisense: UUGUUAUAGAGGAUGAAGGtt; and STAMBP shRNA- 2: sense: CCUCUCAGCUAUCCUUCUAtt, antisense: UAGAAGGAUAGCUGAGAGGtt. The cells were plated at a density of 3 × 10^5^ cells/well and cultured for 24 h. Then, lentivirus was added to the cells at a multiplicity of infection of 10. After transfection for 48 h, we conducted selection with puromycin at a concentration of 1 µg/ml (Selleck Chem, S7417) to obtain successfully transfected cells.

### Plasmids and transfection

The following plasmids were purchased from GeneChem (Shanghai, China): lentivirus plasmid (Ubi-MCS-3FLAG-SV40-puromycin) carrying the full-length human STAMBP CDS (gene ID: 10617) and lentivirus plasmid (CMV-MCS-HA-SV40-Neomycin) carrying the full-length human RAI14 CDS (gene ID: 26064). Cells were plated at a density of 3 × 10^5^ cells/well and cultured for 24 h. Then, lentivirus was added to the cells at a multiplicity of infection of 10. After transfection for 48 h, 1 µg/ml puromycin (Selleck Chem, S7417) and 2 mg/ml G418 (Sigma, 108321-42-2) were used to select for cells with successfully transfected STAMBP and RAI14 plasmids, respectively.

### Western blot analysis

In brief, equal amounts of extracted proteins were prepared with radioimmunoprecipitation assay buffer lysis buffer (Cell Signaling Technology, 9806) and protease inhibitors (Cell Signaling Technology, 5871), and the protein concentration was measured with a BCA assay kit (Thermo Fisher, 23225). The proteins were separated by 12% sodium dodecyl sulfate (SDS)-polyacrylamide Gel Electrophoresis and then transferred to polyvinylidene difluoride (PVDF) membranes. The PVDF membranes were blocked in 5% milk for 1 h, incubated with primary antibodies (1:1000) overnight and probed with peroxidase-conjugated secondary antibodies for 1 h. Protein signals were visualized with ECL detection reagents (Santa Cruz, sc-2048) and exposed to X-ray films (Kodak, USA). TBS-T was used to wash the PVDF membranes after each incubation period.

### MTS assays

Cell viability was evaluated with MTS/CellTiter 96 Aqueous One Solution reagent (Promega, G9242). MDA-MB-231 and BT549 cells were seeded in 96-well plates at a density of 2000–3000 cells/well and cultured overnight. The cells were separately treated as indicated in the figure legends for 24, 48, 72, or 96 h, followed by an analysis by MTS assay. Then, 20 μl of MTS reagent was added to each well in the dark, and the cells were incubated for 2 h. The absorbance of the optical density was read at a wavelength of 490 nm with a microplate reader (Sunrise reader, Mannedorf, Switzerland). Data from three independent experiments were calculated as percentages relative to the control. Cell proliferation was calculated as the fold expansion in comparison to the expression of the control group.

### Wound healing assays

Cell migration was evaluated by wound healing assays. Cells were plated at a density of 2 × 10^5^ cells/well and cultured for 24 h. Cell growth was recorded at 24 h after scratching. Then, the percentage of cell wound healing was analyzed by Image-Pro Plus 7 software. All experiments were performed in, at least, triplicate.

### Transwell assays

Cells were plated at a density of 2 × 10^5^ cells/well and cultured for 24 h. A total of 0.5–1 × 10^5^ cells in FBS-free medium were plated in the upper chamber of Transwell inserts (Corning, 3422), and medium with 10% FBS was added to the lower insert chamber. After 24 h of incubation, the chambers were washed with PBS and fixed with paraformaldehyde. Then, the cells remaining on the upper side of the Transwell inserts were wiped off with a cotton swab. The cells were stained with 1% crystal violet for 5–10 min. All experiments were performed in, at least, triplicate.

### Quantitative RT-PCR

RNA was extracted from cells with RNAiso Plus (TaKaRa, 9108) according to the manufacturer’s instructions. The RNA concentration was adjusted to be equal in each group, and a PrimeScript RT Master Mix kit (TaKaRa, RR036B) was used to synthesize first-strand cDNA. The mRNA levels of STAMBP, RAI14, and GAPDH were measured using real-time quantitative PCR with an SYBR Premix Ex Taq kit (TaKaRa, RR820A). The primers for PCR in this study are listed in Supplementary Table 1.

### Coimmunoprecipitation

Cell lysates were prepared with cell lysis buffer (Cell Signaling Technology, 9803) and protease inhibitors. Each specific antibody was mixed with Dynabeads (Invitrogen, 14311D) and incubated for at least 16 h with rotation. Protein concentrations were adjusted to 2 μg/μl. The mixtures of proteins and Dynabeads containing the required antibodies were incubated with rotation at 4 °C for 1 h. After 3 washes with PBS containing 0.5% Tween 20 (PBS-T), the mixtures were resuspended in an SDS loading buffer. After boiling in a water bath for 10 min and centrifugation at 4 °C for 3 min, the supernatant was collected for further analysis.

### Immunofluorescence analysis

MDA-MB-231 and BT549 cells were plated on chamber slides (Thermo Fisher Scientific, 177402) and cultured for 24 h. After 3 washes with PBS, the cells were fixed with 4% paraformaldehyde for 15 min and permeabilized with 0.1% Triton X-100 for 5 min. Then, 5% bovine serum albumin (Sigma, A8806) was used to block the cells for 30 min. After washing with PBS, the cells were incubated with primary antibodies overnight at 4 °C. After washing with PBS, the cells were incubated with secondary antibodies for 1 h at room temperature in the dark. Finally, the slides were incubated with a mounting medium and DAPI (Abcam, ab104139) and then visualized using fluorescence confocal microscopy.

### Animal studies

The protocol for the care and use of all animals in this study was followed in accordance with the guidelines of the Guangdong Animal Center for the ethical treatment of animals and approved by the Institutional Animal Care and Use Committee of Guangzhou Medical University. The xenograft models were established as previously described^20^. Five-week-old nude BALB/c mice (female) were purchased from Vital River Laboratories (Beijing, China) and bred in the Specific Pathogen Free Animal Center of Guangzhou Medical University. Mice were randomly allocated to each group. MDA-MB-231 cells (2.5 × 10^6^) in 200 µl of PBS were inoculated subcutaneously into the axillary area of nude mice. After a week of inoculation, tumor sizes were recorded every other day. On Day 14 after treatment, tumor xenografts were collected and weighed.

### Immunohistochemistry (IHC)

Tumor xenograft sections were immunostained using a MaxVision kit (Maixin Biol, Fuzhou, China) according to the manufacturer’s instructions. Primary antibodies against RAI14, STAMBP, and Ki67 were used to detect protein expression. The results were analyzed by Image-Pro Plus 7 software. The relative staining intensity was normalized to that of the control group.

### Patient selection

Human breast cancer tissues and adjacent tissues were collected from ten patients at the Affiliated Cancer Hospital and Institute of Guangzhou Medical University. STAMBP and RAI14 expression in ten pairs of paraffin-embedded specimens was measured. Written informed consent was obtained from all the participants, and the study was approved by the Ethics Committee of the Affiliated Cancer Hospital and Institute of Guangzhou Medical University.

### Statistical analysis

The statistical significance between two groups was determined by Student’s *t*-tests. GraphPad Prism 9.0 software was used for statistical analyses, and a *p* value of less than 0.05 was regarded as significant. All data are presented as the mean ± SD of three independent experiments.

## Results

### STAMBP favors TNBC progression

**Fig. 1:**
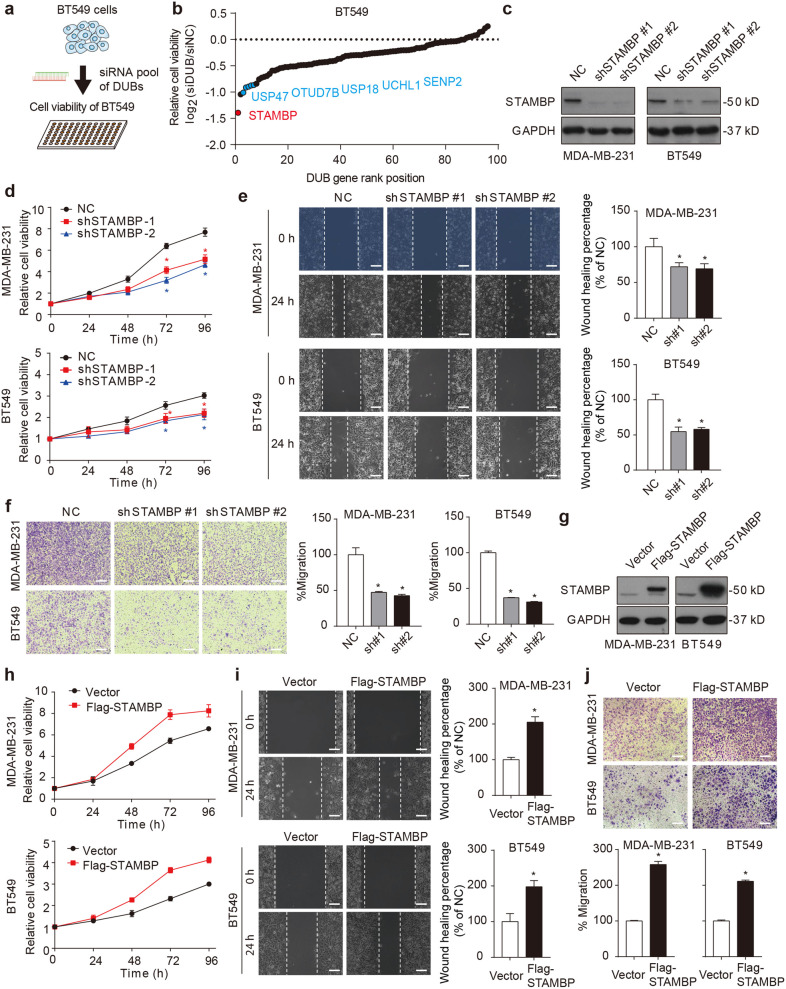
STAMBP promotes proliferation, migration, and invasion of TNBC cells. **a**, **b** Identification of STAMBP as a carcinogenic deubiquitinase in breast cancer cells. BT549 cells were transfected with 96 deubiquitinase siRNAs for 72 h, and cell proliferation was determined by MTS assay. “DUB gene rank position” means ranking DUBs from 1 (lowest) to 96 (highest) according to the relative cell viability, which is calculated by the following formula: log2 (siDUB/siNC ratio). **c** MDA-MB-231 and BT549 cells were stably transfected with two individual STAMBP shRNAs or control shRNA. STAMBP expression in cells was measured by Western blotting. **d**–**f** The proliferation, migration, and invasion of MDA-MB-231 and BT549 cells stably transfected with two individual STAMBP shRNAs or control shRNA for the indicated times were determined by MTS assays (**d**), wound healing assays (**e**), and Transwell migration assays (**f**), respectively. Representative images are shown. Scale bars: 50 μm. The mean ± SD (*n* = 3). *p < 0.05 compared to the NC. **g** MDA-MB-231 and BT549 cells stably expressed vector or Flag-STAMBP. STAMBP protein expression in cells was measured by Western blotting. **h**–**j** Cell proliferation, migration, and invasion of MDA-MB-231 and BT549 cells stably transfected with vector or Flag-STAMBP for the indicated times were determined by MTS assays (**h**), wound healing assays (**i**), and Transwell migration assays (**j**), respectively. Representative images are shown. Scale bars: 50 μm. The wound healing percentage and cell migration percentage with different treatments were quantified with Image-Pro Plus 7 software.

To identify the key DUBs driving TNBC progression, BT549 TNBC cells were transfected with 96 DUB siRNAs for 72 h, and then, cell viability was assayed (Fig. 1a). Among the top positive hits (Fig. 1b and Supplementary Fig. 1), USP47^21^, OTUD7B^22^, USP18^23^, UCHL1^24^, and SENP2^25^ have been previously reported to be associated with breast cancer growth, validating the effectiveness of our screening. Notably, the highest-scoring gene that was essential for the proliferation of BT549 cells encoded STAMBP (Fig. 1b), highlighting a unique role for STAMBP in controlling the growth of TNBC cells.

To assess the contribution of STAMBP to the progression of TNBC, we established two stable STAMBP-knockdown TNBC cell lines (the MDA-MB-231 and BT549 cell lines) and then assayed cell proliferation, migration, and invasion. Consistent with the siRNA screening data, the downregulation of STAMBP (Fig. 1c) significantly inhibited the proliferation of the MDA-MB-231 and BT549 cells (Fig. 1d). The wound healing (Fig. 1e) and Transwell assays (Fig. 1f) further demonstrated that the silencing of STAMBP impaired the migration and invasion of MDA-MB-231 and BT549 cells. The hypothesis that STAMBP mediates tumor cell growth, invasion, and migration was confirmed by the overexpression of STAMBP after gene transfection (Fig. 1g–j). The results of these two-way interventions provide strong evidence supporting the tumor-promoting effect of STAMBP in TNBC.

### STAMBP interacts with RAI14

**Fig. 2:**
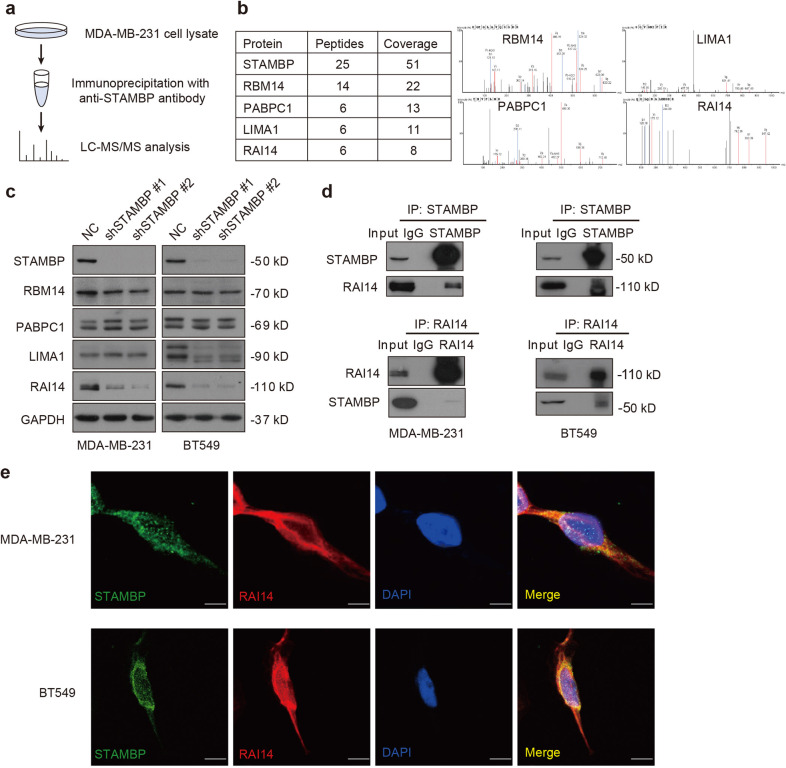
STAMBP interacts with RAI14. **a** MDA-MB-231 cell lysates were incubated with beads conjugated with anti-STAMBP antibodies. Immunoprecipitates were analyzed by mass spectrometry. **b** Ambipolar ion peaks of the indicated proteins are shown. **c** Immunoblot analysis of the indicated protein expression in MDA-MB-231 and BT549 cells stably transfected with two individual STAMBP shRNAs or control shRNA. **d** Endogenous STAMBP and RAI14 interactions were detected by coimmunoprecipitation in MDA-MB-231 and BT549 cells. **e** The cellular location of STAMBP (green) and RAI14 (red) in MDA-MB-231 and BT549 cells was examined by immunofluorescence staining using STAMBP and RAI14 antibodies and visualized by fluorescence microscopy. DAPI (4′,6-diamidino-2-phenylindole) was used to stain nuclei. Scale bars: 10 μm.

Since STAMBP is a DUB, we next used immunoprecipitation in combination with mass spectrometry (IP-MS) to discover substrates for STAMBP involving the TNBC phenotype (Fig. 2a). The IP-MS analysis revealed that four proteins (RBM14, RAI14, LIMA1, and PABPC1) were the top candidates for STAMBP-binding proteins (Fig. 2b). To determine whether STAMBP affects the expression of these candidates, the protein expression of RBM14, RAI14, LIMA1, and PABPC1 was measured by Western blotting in control and STAMBP-knockdown cells. In MDA-MB-231 and BT549 cells, the knockdown of STAMBP resulted in the downregulation of RAI14 but not the downregulation of RBM14 or PABPC1 (Fig. 2c). STAMBP knockdown led to the downregulation of LIMA1 in BT549 cells but not in MDA-MB-231 cells (Fig. 2c), indicating a context-dependent role for STAMBP in the regulation of LIMA1 expression.

To further examine whether RAI14 acts as a direct substrate of STAMBP, we analyzed the interaction between STAMBP and RAI14. Co-IP assays demonstrated the endogenous interaction of STAMBP and RAI14 in MDA-MB-231 and BT549 cells (Fig. 2d). The colocalization of STAMBP and RAI14 was also observed in MDA-MB-231 and BT549 cells via immunofluorescence assays (Fig. 2e). Considering these results, we concluded that STAMBP directly interacts with RAI14 in TNBC cells.

### STAMBP stabilizes RAI14 by decreasing its ubiquitination

**Fig. 3:**
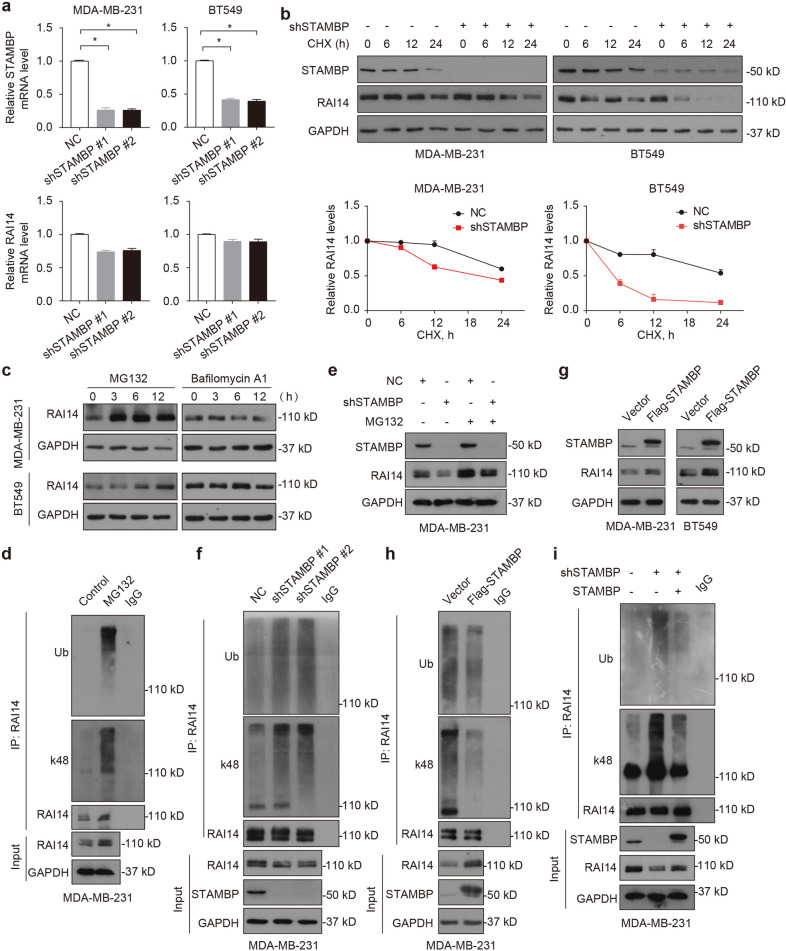
STAMBP deubiquitinates and stabilizes RAI14. **a** qPCR analysis of STAMBP and RAI14 gene expression in MDA-MB-231 and BT549 cells stably transfected with negative control (NC) or STAMBP shRNA. The mean ± SD (*n* = 3), *p < 0.05. **b** MDA-MB-231 and BT549 cells transiently transfected with STAMBP shRNA #1 or control shRNA were treated with cycloheximide (CHX, 100 μg/ml) for the indicated time periods. The protein levels of STAMBP and RAI14 were measured by Western blotting. The intensity of RAI14 expression for each time point was quantified by ImageJ software. The mean ± SD (*n* = 3). *p < 0.05. **c** MDA-MB-231 and BT549 cells were treated with MG132 (10 μM) or bafilomycin A1 (0.1 μM) for the indicated time. The protein levels of RAI14 were measured by Western blotting. GAPDH was used as a loading control. **d** MDA-MB-231 cells were treated with or without MG132 for 6 h. The extracts were immunoprecipitated with anti-RAI14 antibodies and immunoblotted with anti-ubiquitin, anti-K48-linked ubiquitin, and anti-RAI14 antibodies. **e** MDA-MB-231 cells stably transfected with STAMBP shRNA #1 or control shRNA were treated with or without MG132 for 12 h. The expression of RAI14 and STAMBP was assessed. **f** MDA-MB-231 cells stably transfected with two individual STAMBP shRNAs or control shRNA were treated with MG132 for 6 h. The extracts were immunoprecipitated with anti-RAI14 antibodies and immunoblotted with anti-ubiquitin, anti-K48-linked ubiquitin, and anti-RAI14 antibodies. **g** Immunoblotting of STAMBP and RAI14 in MDA-MB-231 and BT549 cells stably expressing vector or Flag-STAMBP. **h** MDA-MB-231 cells stably expressing vector or Flag-STAMBP were treated with MG132 for 6 h. The extracts were immunoprecipitated with anti-RAI14 antibodies and immunoblotted with anti-ubiquitin, anti-K48-linked ubiquitin, and anti-RAI14 antibodies. **i** MDA-MB-231 cells stably expressing STAMBP shRNAs and/or STAMBP expression plasmids were treated with MG132 for 6 h. The extracts were immunoprecipitated with an anti-RAI14 antibody and immunoblotted with the indicated antibody.

To confirm the hypothesis that STAMBP regulates the stability of the RAI14 protein, we first ruled out the possibility that STAMBP affects the expression of RAI14 mRNA. qPCR assays showed that the knockdown of STAMBP exerted little or no effect on the mRNA level of RAI14 in MDA-MB-231 and BT549 cells (Fig. 3a). In contrast, the knockdown of STAMBP accelerated RAI14 protein turnover to a greater extent than control shRNA (Fig. 3b). The UPS and autophagy are the two major protein quality control systems in eukaryotic cells, and they share some common signaling pathways and substrates^26^. To determine which degradation system is critical for the degradation of RAI14 in TNBC cells, the effects of the proteasome inhibitor MG132 and the autophagy inhibitor bafilomycin A1 were analyzed. MG132, but not bafilomycin A1, effectively induced the accumulation of RAI14 protein in MDA-MB-231 and BT549 cells (Fig. 3c), indicating that RAI14 was a substrate in the UPS. Consistently, MG132 increased the ubiquitination rate of RAI14 in MDA-MB-231 cells (Fig. 3d). Importantly, MG132 blocked the degradation of RAI14 protein in STAMBP-knockdown MDA-MB-231 cells (Fig. 3e). These results indicated that STAMBP promoted the protein stabilization of RAI14 in TNBC cells.

Although polyubiquitin chains take different forms, K48-linked ubiquitination chains play a major role in proteasomal degradation^27^. We found that the knockdown of STAMBP increased the total ubiquitination and K48-linked ubiquitination rates of RAI14 in MDA-MB-231 cells (Fig. 3f), supporting a key role for STAMBP in RAI14 deubiquitination. In contrast, overexpression of STAMBP increased RAI14 protein expression (Fig. 3g) and decreased the ubiquitination rate of RAI14 in MDA-MB-231 cells (Fig. 3h). As expected, overexpression of STAMBP rescued the ubiquitination of RAI14 caused by STAMBP knockdown (Fig. 3i). However, overexpression of STAMBP with a catalytically inactive site (D348A) failed to decrease the ubiquitination of the RAI14 protein (Supplementary Fig. 2), supporting the hypothesis that STAMBP DUB activity is required for RAI14 deubiquitination. Collectively, these findings indicated that STAMBP stabilized RAI14 by decreasing its ubiquitination.

### RAI14 is required for STAMBP-mediated TNBC growth

**Fig. 4:**
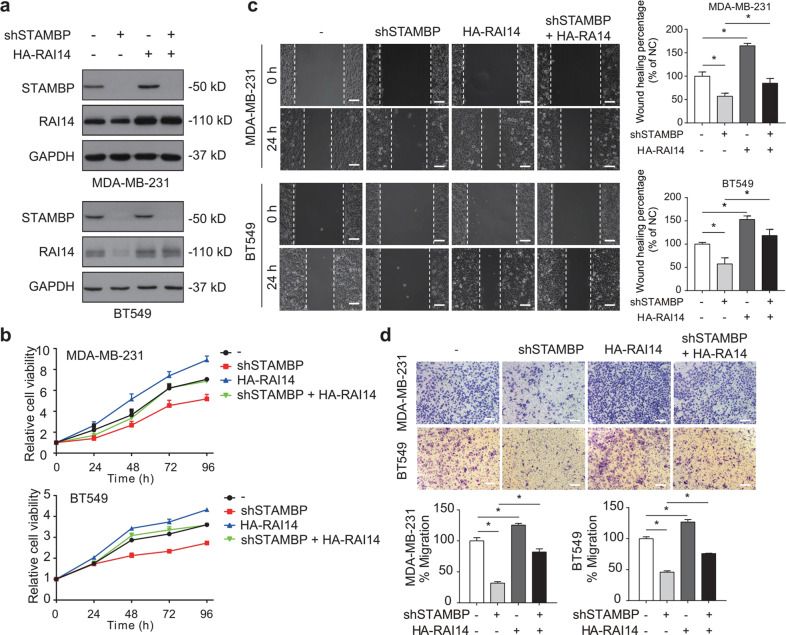
STAMBP promotes TNBC progression by regulating RAI14. **a** MDA-MB-231 and BT549 cells stably transfected with STAMBP shRNA #1 or control shRNA were stably expressed with HA-RAI14 or vector. STAMBP and HA-RAI14 expression in cells was measured by Western blotting. **b**–**d** Cell proliferation, migration, and invasion of the indicated MDA-MB-231 and BT549 cells were determined by MTS assays (**b**), wound healing assays (**c**), and Transwell migration assays (**d**), respectively. Representative images are shown. Scale bars: 50 μm. The mean ± SD (*n* = 3). *p < 0.05.

**Fig. 5:**
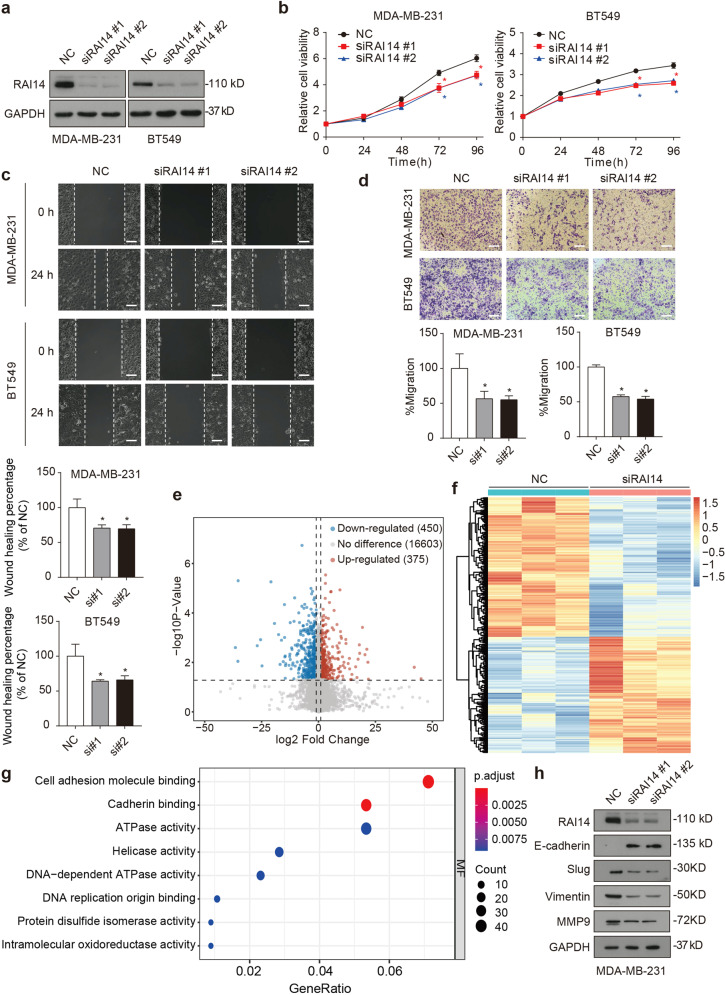
RAI14 promotes cell proliferation, migration, and invasion of TNBC. **a** MDA-MB-231 and BT549 cells were transfected with two independent RAI14 siRNAs or control siRNAs. RAI14 expression in cells was measured by Western blotting. **b**–**d** Cell proliferation, migration, and invasion of MDA-MB-231 and BT549 cells transfected with two independent RAI14 siRNAs or control siRNA were determined by MTS assays (**b**), wound healing assays (**c**), and Transwell migration assays (**d**), respectively. Representative images are shown. The wound healing percentage and migration percentage were determined with Image-Pro Plus 7 software. **e**–**g** RNA-seq analysis in MDA-MB-231 cells following siRNA #1-mediated knockdown of RAI14. The mean ± SD (*n* = 3). *p < 0.05 compared to the NC. **e** Volcano plot of gene expression (siRAI14 versus the siControl; fold change, ≥2; *p* < 0.05) between RAI14 knockdown and control MDA-MB-231 cells. **f** Heatmap of differential gene expression between RAI14 knockdown and control MDA-MB-231 cells. **g** GO analysis of differentially expressed genes between RAI14 knockdown and control MDA-MB-231 cells. **h** MDA-MB-231 cells were transfected with two independent RAI14 siRNAs or control siRNAs. The expression of the indicated proteins in cells was measured by Western blotting.

We next asked whether RAI14 plays a key role in STAMBP-mediated TNBC progression. The HA-RAI14 plasmid was stably expressed in control or STAMBP-knockdown MDA-MB-231 and BT549 cells (Fig. 4a). The overexpression of RAI14 released the inhibition on cell proliferation (Fig. 4b), cell migration (Fig. 4c) and cell invasion (Fig. 4d) in STAMBP-knockdown cells. To further characterize the role of RAI14 in TNBC cells, we used two nonoverlapping siRNAs to suppress RAI14 expression in MDA-MB-231 and BT549 cells (Fig. 5a). We observed that phenotypes in the RAI14-knockdown cells were similar to those in STAMBP-knockdown cells in terms of proliferation (Fig. 5b), migration (Fig. 5c), and invasion (Fig. 5d). In addition, STAMBP was stably overexpressed in control and RAI14-knockdown MDA-MB-231 and BT549 cells (Supplementary Fig. 3a). Overexpression of STAMBP released RAI14 knockdown-induced inhibition of cell proliferation (Supplementary Fig. 3b), cell migration (Supplementary Fig. 3c) and cell invasion (Supplementary Fig. 3d). Overall, these results indicated that RAI14 was a downstream effector of STAMBP that maintained the TNBC phenotype.

To further illuminate the underlying mechanisms of the downstream effect of the STAMBP-RAI14 pathway in TNBC cells, we performed an RNA sequencing analysis in control and RAI14-knockdown cells. Compared with control cells, there were 450 downregulated genes and 375 upregulated genes in the RAI14-knockdown cells (fold change ≥2; *p* < 0.05) (Fig. 5e). A subsequent comparison of gene expression differences (Fig. 5f) and a Gene Ontology (GO) analysis revealed that the knockdown of RAI14 affected multiple pathways, especially cell adhesion molecule-binding activity in molecular function (Fig. 5g), which is closely related to malignant transformation, cancer cell migration, and tumor metastasis^28^. A similar trend was observed in the STAMBP-knockdown cells, which showed altered expression of most cell adhesion-related genes (25/40), and these changes were reversed by RAI14 overexpression (Supplementary Fig. 4). It has been reported that cell adhesion changes play an important role in regulating the epithelial-to-mesenchymal transition (EMT)^29^. EMT is known to maintain cancer development and metastasis; therefore, we further examined whether RAI14 regulates the EMT in TNBC. We found that knocking down RAI14 increased the levels of epithelial markers, such as E-cadherin, and decreased those of mesenchymal markers, such as slug, vimentin, and MMP9, in MDA-MB-231 cells (Fig. 5h). Similarly, knocking down STAMBP also inhibited EMT-like changes, and this effect was reversed by overexpression of RAI14 (Supplementary Fig. 5). Taken together, these findings demonstrated that RAI14 was critical for the STAMBP-mediated cell growth of TNBC cells.

### The STAMBP-RAI14 pathway drives tumor growth in vivo

**Fig. 6:**
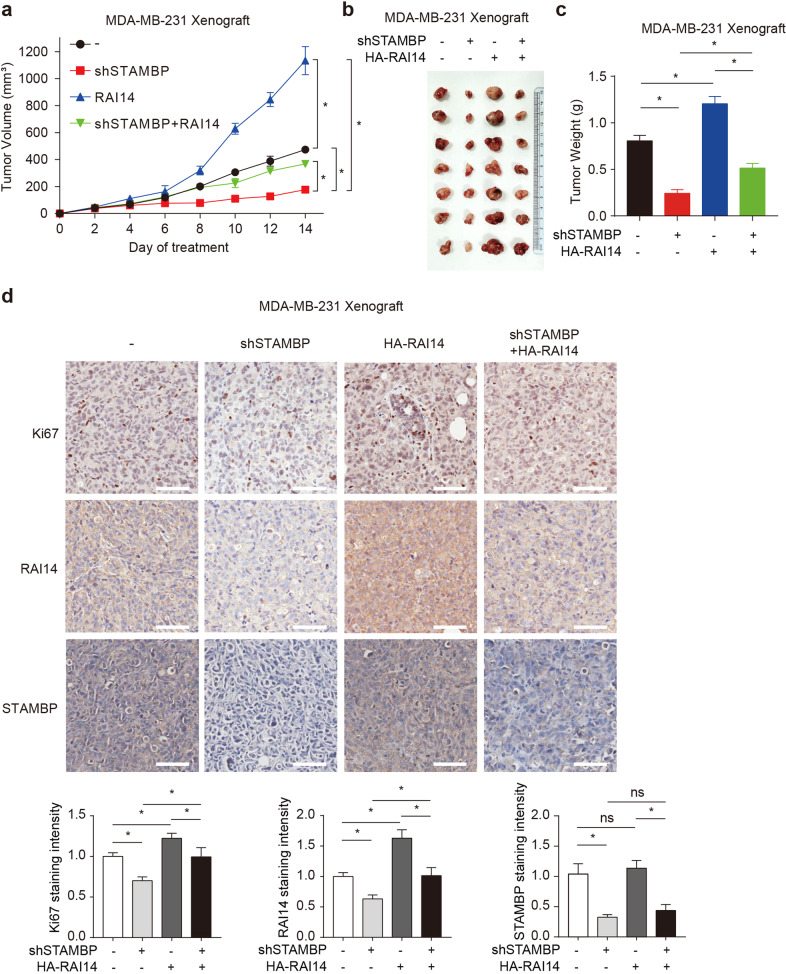
The STAMBP-RAI14 pathway regulates the development of TNBC in vivo. **a**–**c** Tumor xenografts were established in BALB/c nude mice using MDA-MB-231 cells stably transfected with STAMBP shRNA #1 or overexpressing HA-RAI14. Tumor size was recorded every other day. Tumor size (**a**), tumor images (**b**), and tumor weight (**c**) are shown (7 mice/group). The mean ± SD (*n* = 7), *p < 0.05. **d** Representative image of immunohistochemical staining for Ki67, RAI14, and STAMBP in nude mouse tumor tissues (200×). Scale bars: 50 μm. Ki67, RAI14, and STAMBP staining intensities were quantified with Image-Pro Plus 7 software. Mean ± SD (*n* = 7). *p < 0.05. ns, not significant.

To study the effect of the STAMBP-RAI14 pathway on tumor growth in vivo, we injected control or STAMBP-knockdown MDA-MB-231 cells stably expressing HA-RAI14 into the axillary area of BALB/c nude mice to establish a xenograft model. The STAMBP-knockdown group showed reduced xenograft tumor growth (Fig. 6a), which was accompanied by a reduction in tumor volume (Fig. 6b) and tumor weight (Fig. 6c). Similar to the protumor effect of RAI14 in vitro, the overexpression of RAI14 reversed the xenograft tumor reduction caused by the knockdown of STAMBP to a certain extent (Fig. 6a–c). Furthermore, the protein level of the proliferation marker Ki67 was significantly decreased by STAMBP knockdown, but this effect was reversed by RAI14 overexpression (Fig. 6d). These animal results demonstrated that STAMBP promoted TNBC progression partially by sustaining RAI14 expression in vivo.

### STAMBP expression predicts poor prognosis in TNBC patients

**Fig. 7:**
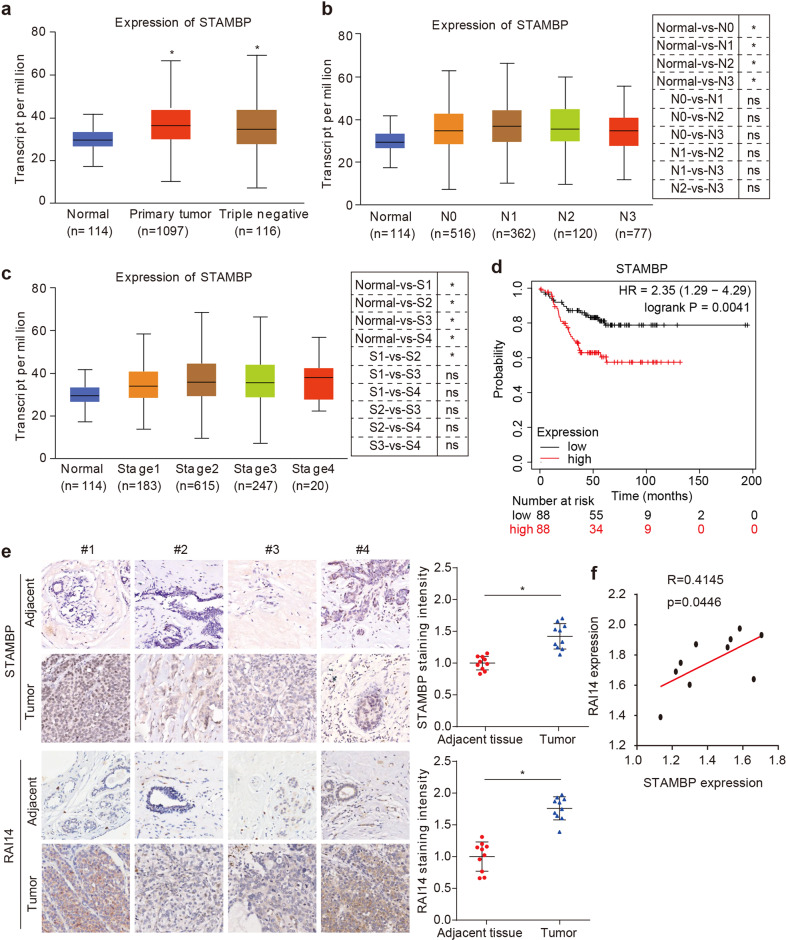
STAMBP expression predicts poor prognosis in TNBC. **a** Boxplot showing the relative expression of STAMBP in normal and breast cancer samples using the UALCAN web resource. **b** Box plot showing the relative expression of STAMBP in breast cancer based on nodal metastasis status using the UALCAN web resource. **c** Box plot showing the relative expression of STAMBP in breast cancer based on individual cancer stages using the UALCAN web resource. The mean ± SD. *p < 0.05. ns, not significant. **d** Association between STAMBP expression and breast cancer patient survival. Data analyses were performed using Kaplan‒Meier Plotter. **e** Representative images of STAMBP or RAI14 expression in human TNBC tissues (*n* = 10) and adjacent tissues (*n* = 10). Scale bars: 50 μm. The STAMBP or RAI14 staining intensity was quantified with Image-Pro Plus software (right graph). The mean ± SD (*n* = 10). *p < 0.05. **f** The correlation of STAMBP and RAI14 expression in the tumor (*n* = 10).

To further explore the clinical relevance of STAMBP expression in human breast cancer, we used the UALCAN database (http://ualcan.path.uab.edu/)^30^ to investigate the mRNA level of STAMBP in breast tumor tissues with respect to lymph node metastasis status and individual cancer stages. Compared with normal tissues, the expression of STAMBP was significantly higher in human breast tumor tissues, especially in human TNBC tissues (Fig. 7a). Moreover, compared with that in normal tissues, the level of STAMBP mRNA in breast cancer tissues of breast cancer patients with or without lymph node metastasis was increased (Fig. 7b). However, no significant difference was found between the patients with (N1/N2/N3) and without (N0) lymph node metastasis (Fig. 7b). In addition, compared with that in normal tissue, the level of STAMBP mRNA in breast cancer tissues in patients with Stage 1, 2, 3, or 4 breast cancer was increased (Fig. 7c). STAMBP showed higher expression in advanced clinical Stage 2 than in Stage 1 (Fig. 7c). However, the level of STAMBP did not significantly differ between the other stage groups. These findings confirm that STAMBP protein expression was highly expressed in tumor tissue from breast cancer patients.

We then used publicly available databases (www.kmplot.com)^31^ to evaluate the prognostic effect of STAMBP mRNA expression in TNBC patients. TNBC patients with higher STAMBP mRNA expression showed a lower overall survival (Fig. 7d). To investigate whether STAMBP and RAI14 proteins show similar clinical significance, we analyzed their protein expression levels in human TNBC and adjacent tissues (*n* = 10) via immunohistochemical assay. Compared with that in adjacent tissues, the protein expression of STAMBP or RAI14 was upregulated in human TNBC tissues (Fig. 7e). In addition, STAMBP protein expression was positively correlated with RAI14 protein expression in human TNBC tissues (Fig. 7f). These bioinformatics and immunohistochemical analyses indicated that the upregulation of STAMBP was related to the development of TNBC.

## Discussion

**Fig. 8:**
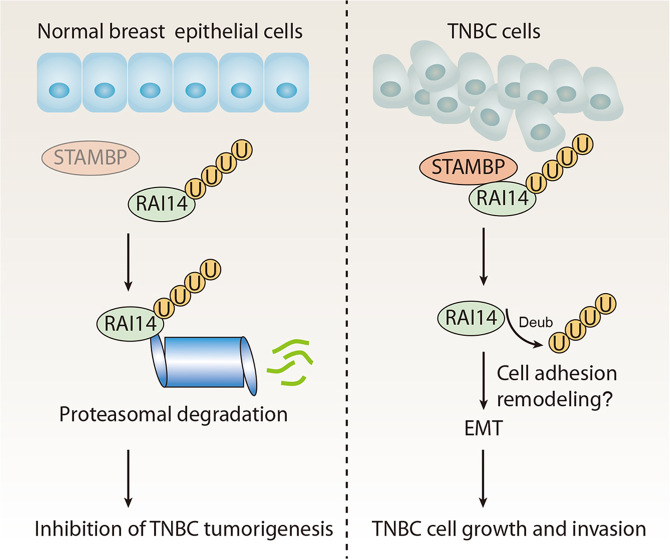
A working model of STAMBP action in TNBC. STAMBP, which is highly expressed in TNBC, exerts its oncogenic effect by inhibiting RAI14 ubiquitination and enhancing RAI14 stabilization, thereby possibly promoting cell adhesion remodeling and EMT in TNBC.

Due to the lack of three known therapeutic targets (ER, PR, and Her-2), TNBC is a challenging female tumor to study and treat. An in-depth understanding of the growth and invasion signaling in TNBC may lead to the development of new therapeutic targets. In this study, our unbiased siRNA and substrate screening revealed a previously unknown role of STAMBP in TNBC: STAMBP direct removes ubiquitin from RAI14, enhancing the stability of tumor-promoting RAI14 (Fig. 8). Since STAMBP and RAI14 are overexpressed in TNBC, our findings may establish a novel strategy to inhibit TNBC growth and invasion.

DUBs are proteases that cleave ubiquitin moieties from substrates and have been implicated in various pathways of tumor biology^32^. Accordingly, DUBs are key therapeutic targets for cancer therapy. Compared with that in other cancers, the direct role of DUB in TNBC is still poorly understood. In the current study, we proved that STAMBP is the most significant DUB gene associated with the growth of TNBC. Our analysis of public datasets and clinical specimens provides further support for findings suggesting that STAMBP may contribute to the tumor progression of TNBC. Performing functional experiments, we further demonstrated that the upregulation of STAMBP promoted the proliferation and invasion of TNBC cells, while the knockdown of STAMBP suppressed the malignant progression of TNBC cells. Previous studies have shown that STAMBP promotes the migration and invasion of lung cancer^18^ and melanoma cells^19^. In line with these studies, we provide the first evidence that STAMBP is a potential target for the treatment of TNBC.

Identifying tumor-related protein substrates is a challenge in UPS applications. Early studies have shown that STAMBP participates in the deubiquitination of several proteins, including NALP7^15^, NLRP3^16^, ULK1^17^, and EGFR^18^, under different conditions. Our IP–MS analysis revealed that RAI14 is a common protein-binding partner of STAMBP in TNBC cells. Subsequent experiments showed that RAI14 is a substrate of the UPS and that its ubiquitination and proteasomal degradation are regulated by STAMBP. RAI14, also known as NORPEG, was first found in human retinal pigment epithelial cells (ARPE-19), and it was found to be induced by all-trans-retinoic acid^33^. In addition to its physiological functions, dysfunctional RAI14 contributes to the development of multiple cancers, including glioblastoma^34^, gastric cancer^35^, melanoma^36^, esophageal cancer^37^, and breast cancer^38^. Our in vitro and in vivo experiments not only showed that RAI14 contributes a phenotype similar to that associated with STAMBP but also demonstrated that RAI14 overexpression reversed the inhibition of proliferation and migration that had been induced by STAMBP knockdown. Although our data establish a new STAMBP-RAI14 pathway, the effects of other known STAMBP substrates in TNBC require further study.

Our RNA sequencing, in combination with GO enrichment analysis, indicated that RAI14 is closely associated with the cell adhesion pathway. Homeostasis in healthy tissues greatly depends on cell‒cell adhesion and cell–extracellular matrix interactions^39^. In addition, cell adhesion plays a key role in the development of tumor recurrence, invasiveness, and distant metastasis^28^. RAI14 is a member of the ankycorbin family and is related to the cortical actin cytoskeleton structure, cell‒cell adhesion sites, and stress fibers^40^. Our results indicate that STMABP may drive the proliferation and invasion of TNBC by regulating the RAI14-mediated cell adhesion pathway, thereby providing a theoretical basis for the potential targeting of RAI14 protein degradation in the treatment of metastatic breast cancer.

Studies have shown that pharmacologic inhibition of DUBs exerts a significant antineoplastic effect on various tumor cells^8^. For example, the proteasomal DUB (USP14 and UCHL5) inhibitor VLX1570 has been entered in phase I trials for the treatment of multiple myeloma^41,42^. BC-1471 is a small-molecule inhibitor against the catalytic activity of STAMBP, which may reduce the level of its substrate NALP7 protein in THP-1 cells^15^. However, other studies have shown that BC-1471 exerted no inhibitory effect on STAMBP-mediated ubiquitination deconjugation^43^ or STAMBP-mediated EGFR stabilization in vitro^18^. Similarly, we found that BC-1471 exerted no obvious effects on the viability of MDA-MB-231 cells (Supplementary Fig. 6a) or the degradation of RAI14 (Supplementary Fig. 6b) at the reported effective concentration. Nevertheless, specific STAMBP inhibitors need to be developed before the translation of targetable STAMBP into medical applications for cancer is realized.

In summary, our results revealed a previously unrecognized molecular link between UPS and TNBC mediated through STAMBP. Further understanding of STAMBP-dependent RAI14 protein stability may guide the development of improved strategies for the treatment of metastatic cancer, including TNBC.

## Supplementary information

Supplementary information
