# Supplementary material for: The deubiquitinating enzyme STAMBP is a newly discovered driver of triple-negative breast cancer progression that maintains RAI14 protein stability
Source: Exp Mol Med. 2022 Nov 25;54(11):2047–59. doi: 10.1038/s12276-022-00890-1 (PMC9723177; doi:10.1038/s12276-022-00890-1)
Supplement: Supplementary file 1 — Supplementary information [file 12276_2022_890_MOESM1_ESM.pdf]

The deubiquitinating enzyme STAMBP is a newly discovered driver of triple-negative breast cancer progression that maintains RAI14 protein stability

Supplementary figures and figure legends

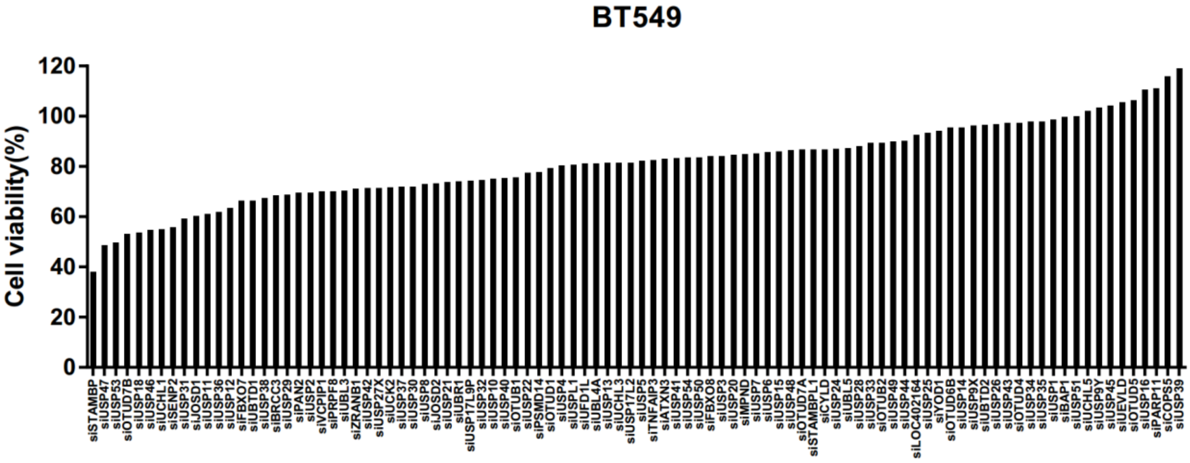

Supplementary Fig. 1 Effects of knockdown of DUBs on cell proliferation of BT549 cells. BT549 cells were transfected with 96 deubiquitinase siRNA respectively for 72 h, and then cell proliferation was determined by MTS assay.

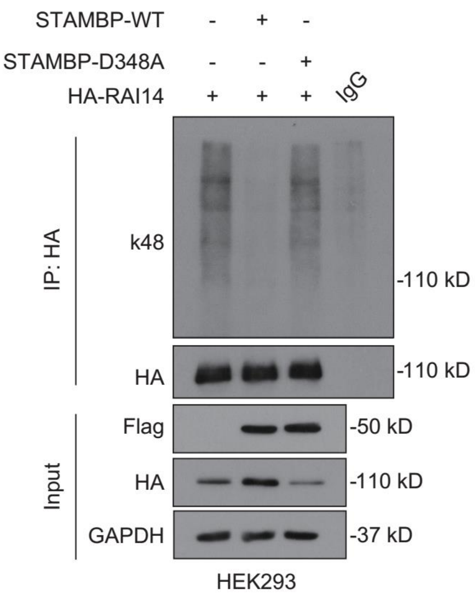

Supplementary Fig. 2 STAMBP D348A mutant fails to deubiquitinate RAI14.

HEK293 cells transiently transfected with HA-RAI14, STAMBP-WT or STAMBP-D348A plasmid were treated with MG132 for 6 h. The extracts were immunoprecipitated with anti-HA antibody and immunoblotted with the indicated antibody.

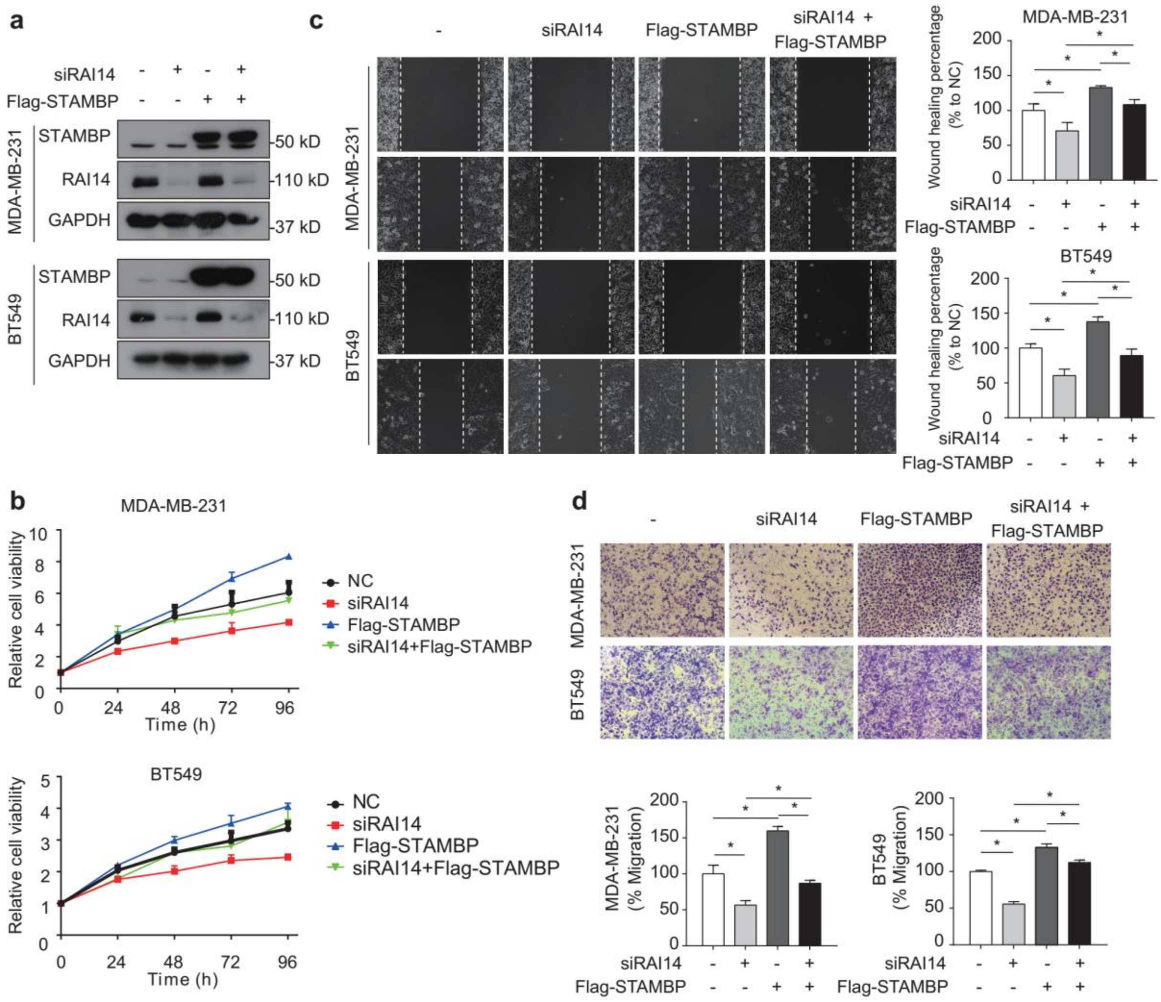

Supplementary Fig. 3 STAMBP-RAI14 pathway promotes cell proliferation, migration, and invasion of TNBC.

a MDA-MB-231 and BT549 cells stably transfected with flag-STAMBP were transfected with RAI14 siRNA or control siRNAs. RAI14 and STAMBP expression in cells was measured by Western blot. **b-d** Cell proliferation, migration, and invasion of the indicated MDA-MB-231 and BT549 cells were determined by MTS assays **(b)**, wound healing assays **(c)**, and transwell migration assays **(d)**, respectively. Representative images are shown. Wound healing percentage and migration percentage were countered with Image-Pro Plus 7 software. Mean ± SD (n = 3). *$p < 0.05$.

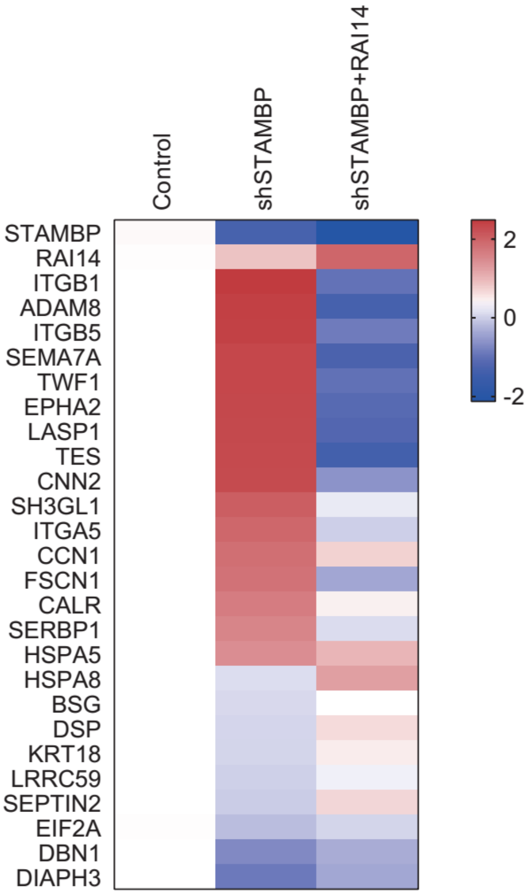

Supplementary Fig. 4 STAMBP regulates cell adhesion pathway by regulating RAI14. MDA-MB-231 were stably transfected with STAMBP shRNA #1 or HA-RAI14. Expression of the indicated genes in cells was measured by qPCR analysis. Gene expression levels are normalized to control cells (100%).

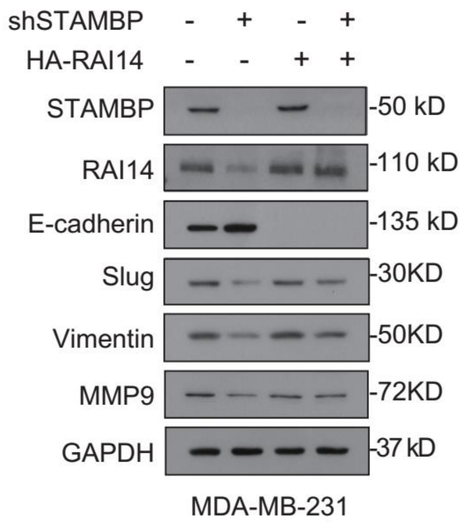

Supplementary Fig. 5 STAMBP promotes EMT by regulating RAI14.

MDA-MB-231 cells stably transfected with STAMBP shRNA #1 or control shRNA were stably expressed with HA-RAI14 or vector. Expression of the indicated proteins was measured by Western blot.

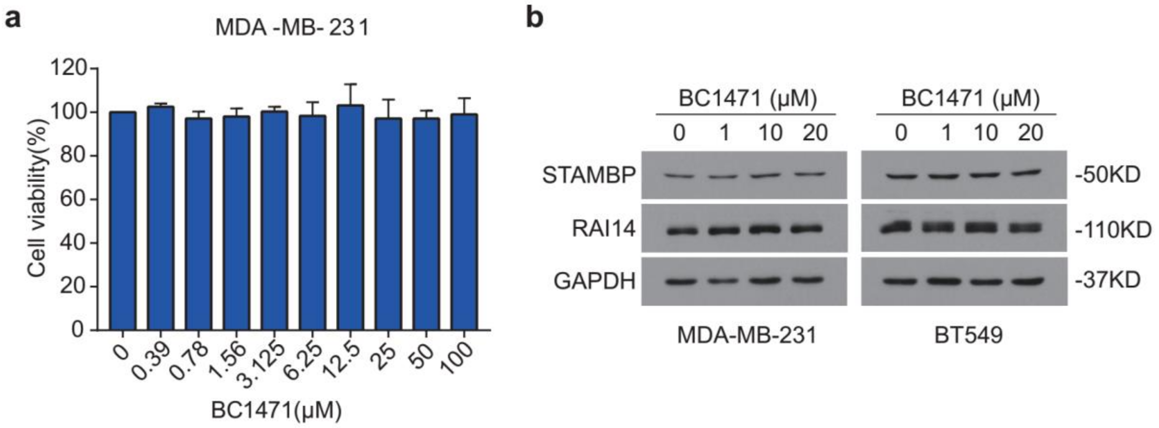

Supplementary Fig. 6 Effects of BC1471 in TNBC cells.

a MDA-MB-231 cells were treated with the indicated doses of BC1471 for 72 h, and then cell proliferation was determined by MTS assay. **b** MDA-MB-231 and BT549 cells were treated with the indicated doses of BC1471 for 24 h. The protein levels of STAMBP and RAI14 were measured by Western blot.

Supplementary Table 1 The primer sequences used for PCR in this study.

Gene name	Forward Sequence	Reverse Sequence
STAMBP	CGGTAGAGGTGAATGAAGACAT	TGTTGCCTTCCTCAGAGTAAAT
RAI14	GGCAGAACTGGTATGCTTAAAC	TCCTCGTATTTGCTCTGAGTTT
GAPDH	TCCCATCACCATCTTCCA	CATCACGCCACAGTTTCC
ITGB1	GGATTCTCCAGAAGGTGGTTTCG	TGCCACCAAGTTTCCCATCTCC
ADAM8	TGCTGGAGGTGGTGAATCACGT	TCAGGAGGTTCTCCAGTGTGAC
ITGB5	GCCTTTCTGTGAGTGCGACAAC	CCGATGTAACCTGCATGGCACT
SEMA7A	CTTCTTCCGAGAGGACAATCCTG	GTGTTCCACTTGGAGACTGACAG
TWF1	CCCAAGGATTCAGCTCGTTACC	GCTAGAATACAGCATCCGCTCTC
EPHA2	ACTGCCAGTGTCAGCATCAACC	GTGACCTCGTACTTCCACACTC
LASP1	CTTCGCCTCAAGCAACAGAGTG	TGTCTGCCACTACGCTGAAACC
TES	GTGGCAGACATTACTGTGACAGC	CAGCAGAAGTGTTTCAGGTGCC
CNN2	GGTGGACATTGGCGTCAAGTAC	GGGTCATAGAGATGCCTTCTCG
SH3GL1	CGGCGAGTCCAACTTTGGTGAC	CTCCTTCAGGTCTTTCTCGCAC
ITGA5	GCCGATTCACATCGCTCTCAAC	GTCTTCTCCACAGTCCAGCAAG
CCN1	GGAAAAGGCAGCTCACTGAAGC	GGAGATACCAGTTCCACAGGTC
FSCN1	GACACCAAAAAGTGTGCCTTCCG	CAAACTTGCCATTGGACGCCCT
CALR	TCAAGGAGCAGTTTCTGGACGG	GCATCCTGGCTTGTCTGCAAAC
SERBP1	AGAAAGGCGACCACCTCGTGAA	ACCTCTTCCAAGACCACCACGA
HSPA5	CTGTCCAGGCTGGTGTGCTCT	CTTGGTAGGCACCACTGTGTTC

HSPA8	TCCTACCAAGCAGACACAGACC	CAGGAGGTATGCCTGTGAGTTC
BSG	GGCTGTGAAGTCGTCAGAACAC	ACCTGCTCTCGGAGCCGTTCA
DSP	TGACAGACCGCTGGCAAAGGAT	GGCGTTTAGCATCATAGAGCCAC
KRT18	GCTGGAAGATGGCGAGGACTTT	TGGTCTCAGACACCACTTTGCC
LRRC59	TCCAGCACCTGGATCTCCTCAA	TCATCCAAGCAGTCACCTGCCA
SEPTIN2	GGTGACGCTATCAACTGCAGAG	ATGATGTGCCGCCTGTTCAAGC
EIF2A	CTGGACCTCATGCAGCTTTAGC	CTCCATAGTAGGAAGCTCCTGTC
DBN1	CACCTACCAGAAGACGGATGCA	TGCTCGAACCTGAGCCTCTCAT
DIAPH3	GATGAAACACGGTTGGCAGAGTC	ACTGCTCAGGTTCACATAAGTTGC
